# Controlling Bacterial Pathogens in Water for Reuse: Treatment Technologies for Water Recirculation in the Blue Diversion Autarky Toilet

**DOI:** 10.3389/fenvs.2017.00090

**Published:** 2017-12-19

**Authors:** Mi T. Nguyen, Lukas Allemann, Christopher Ziemba, Odile Larive, Eberhard Morgenroth, Timothy R. Julian

**Affiliations:** 1 Eawag, Swiss Federal Institute of Aquatic Science and Technology, Dübendorf, Switzerland; 2 Nguyen Tat Thanh Hi-Tech Institute, Nguyen Tat Thanh University Ho Chi Minh City, Vietnam; 3 ETH Zürich, Institute of Environmental Engineering, Zurich, Switzerland; 4 EPFL, School of Architecture, Civil and Environmental Engineering, Lausanne, Switzerland

**Keywords:** water for reuse, pathogen, inactivation, regrowth, biologically active membrane bioreactor, biostability

## Abstract

The Blue Diversion AUTARKY Toilet is a urine-diverting toilet with on-site treatment. The toilet is being developed to provide a safe and affordable sanitation technology for people who lack access to sewer-based sanitation. Water used for personal hygiene, hand washing, and flushing to rinse urine- and feces-collection bowls is treated, stored, and recycled for reuse to reduce reliance on external water supplies. The system provides an opportunity to investigate hygiene of water for reuse following treatment. Treatment in the toilet includes a Biologically Activated Membrane Bioreactor (BAMBi) followed by a secondary treatment technology. To identify effective secondary treatment, three options, including granular activated carbon (GAC) only, GAC+chlorine (sodium hypochlorite), and GAC+electrolysis are considered based on the bacterial inactivation and growth inhibition efficiency. Four different hygiene-relevant bacteria are tested: *Escherichia coli, Enterococcus faecalis, Pseudomonas aeruginosa*, and *Salmonella typhimurium*. Our evaluation demonstrates that—despite treatment of water with the BAMBi—*E*. *coli, P aeruginosa*, and *S. typhimurium* have the potential to grow during storage in the absence of microbial competition. Including the indigenous microbial community influences bacterial growth in different ways: *E. coli* growth decreases but *P. aeruginosa* growth increases relative to no competition. The addition of the secondary treatment options considerably improves water quality. A column of GAC after the BAMBi reduces *E. coli* growth potential by 2 log_10_, likely due to the reduction of carbon sources. Additional treatments including chlorination and electrolysis provide further safety margins, with more than 5 log-_10_ inactivation of *E. coli*. However, reactivation and/or regrowth of *E. coli* and *P. aeruginosa* occurs under in the absence of residual disinfectant. Treatment including the BAMBi, GAC, and electrolysis appear to be promising technologies to control bacterial growth during storage in water intended for reuse.

## Highlight

- Bacterial growth in fecally-contaminated water is highly variable and dependent on several factors.
- Regrowth occurs after chlorination (low doses, no residual).
- Indigenous microbial communities variably impact bacterial growth.
- A combination of treatments can both inactivate and inhibit growth.

## INTRODUCTION

Two-thirds of the world’s population suffer from water scarcity (United Nations, 2012; Global Water Institute, 2013; Mekonnen and Hoekstra, 2016). Water recovery and reuse from diverse sources (i.e., graywater, wastewater, stormwater) can help to increase water efficiency and reduce impacts of scarcity. However, reuse may pose risks to environmental and human health, especially when source water is fecally-contaminated (Christova- Boal et al., 1996; Gross et al., 2005; Wiel-Shafran et al., 2006). Fecally-contaminated water (water containing feces, such as blackwater, brownwater, and wastewater) may contain high concentrations of microbial contamination, including fecal bacteria (e.g., *E. coli*, enterococci), enteric pathogens (e.g., *Salmonella *typimurium*, Cryptosporidium spp., Giardia* spp.), and/or opportunistic pathogens (e.g., *Pseudomonas aeruginosa*) (Christova-Boal et al., 1996; Albrechtsen, 2002; O’Toole et al., 2012; Katukiza et al., 2015). Exposures during reuse like inhaling aerosols generated from toilet flushing, indirect ingestion via hand-to-mouth contacts, unintentional ingestion (Christova- Boal et al., 1996), or consumption of plants irrigated using fecally- contaminated water (Shuval et al., 1997; Mara et al., 2007) may contribute to disease transmission (Morel and Diener, 2006).

Risks from fecally-contaminated water reuse can be mitigated through safe management, including chemical and physical disinfection. Disinfection reduces the concentration of pathogens in water and helps prevent pathogen growth during subsequent distribution and/or storage. Chlorine disinfection (1.4 mg L^-1^ with 30 min exposure, or 42 mg min L^-1^) was shown to be effective in inactivating fecal coliforms in treated graywater, with no regrowth (or growth following disinfection) (Friedler et al., 2006). Membrane filtration (e.g., MBR, ultrafiltration) used to treat graywater achieved up to 4 log10 removal of fecal coliforms (Friedler et al., 2006). UV irradiation (25-40 mJ cm^-2^, unreported water thickness) was also shown to be effective in lowering total coliforms (to 2-500 CFU/100mL) and fecal coliforms (to 2-30 CFU/100mL) to meet German quality guidelines for graywater reuse (Nolde, 2000). In another study, Gilboa and Friedler (2008) report observing no regrowth of fecal coliforms,* P. aeruginosa, or Staphylococcus aureus* after exposure to UV disinfection (0-439 mJ cm^-2^, unreported water thickness) up to 6h (Gilboa and Friedler, 2008). Although treatment processes can significantly reduce the concentration of bacteria, regrowth can occur in treated fecally-contaminated water during storage and distribution due to the availability of assimilable organic carbon (AOC) and the loss of disinfectant residual (Jjemba et al., 2010; Thayanukul et al., 2013; Lin et al., 2016). Other factors can affect the growth of pathogens in disinfected water, including type and concentration of available nutrients, type and concentration of residual disinfectant, presence of indigenous community, water age, pipe materials, and environmental conditions (Wang et al., 2014; Prest et al., 2016).

In this study, risks from reuse of fecally-contaminated water in the Blue Diversion Autarky Toilet (BDAT, http://www.autarky. ch) were investigated. The BDAT was developed by Eawag (Dubendorf, Switzerland) and designed by EOOS (Vienna, Austria) to provide a sanitation option that is safe, affordable, and off-the-grid (Larsen et al., 2015). As a source-separating toilet, the BDAT has urine and feces separately collected and treated for resource recovery. The BDAT also provides and recycles running water, with a design flow rate of 75 L/day for 10 users (Larsen et al., 2015). Despite the source separation strategy, between 1 and 2% of the urine and feces produced by the users enter into the water recycling system. After the BDAT is primed with water from local sources, the water is filtered through a biologically activated membrane bioreactor (BAMBi) composed of aerated flatsheet polyethersulfone membranes with a nominal cutoff of 150 kDa. The BAMBi achieves low, but stable, gravity-driven flux, with the stability attributed to biological activation of the membrane surface (Kunzle et al., 2015). After filtration through the BAMBi, permeate water is stored in a 30 L clean water tank to be used for toilet flushing, hand washing, and personal hygiene before being collected and recirculated back to the BAMBi. This permeate water still contains 40-50 mg L^-1^ of dissolved organic carbon (DOC). The addition of a granular activated carbon (GAC) filter into the top of the clean water tank has demonstrated the ability to reduce this DOC concentration significantly without impacting system operation. The compatibility between permeate water either with or without the GAC treatment and any pathogens that may enter the system is not well-understood, and therefore represents a potential obstacle to the safe utilization of the BDAT.

The specific goal of this study was to evaluate bacterial growth, inactivation, and reactivation/regrowth in treated water during storage within the BDAT. Growth is defined, here, as an increase in cell concentration by either total cell count (TCC), or colony forming units (CFU). Reactivation or regrowth is defined, here, as growth following an observed decrease in cell concentration due to treatment processes. Two fecal indicators (*Escherichia coli and Enterococcus faecalis*) and two pathogens (*Pseudomonas aeruginosa* and *Salmonella typhimurium*) were chosen to study behaviors of waterborne pathogens. Bacterial concentrations were measured using culture method (spreading on selective- media plates) and flow cytometry [TCC and intact cell counts (ICC)]. Results from this study improve the understanding of inactivation and growth/regrowth of fecal indicators and pathogens in water for reuse in the context of the BDAT and other water recycling systems.

## METHODS

Two sets of experiments were performed: (1) growth potential assays to assess bacterial growth within stored water, and disinfection assays to assess bacterial inactivation and reactivation. Both sets of experiments were performed using aliquots of water collected from within the BDAT following treatment by either the BAMBi alone or the BAMBi and the GAC (BAMBi+GAC).

### Blue Diversion AUTARKY Toilet

Water for the experimental assays was collected from full-scale BDATs using recreated influent to mimic expected flush water, hand wash water, and personal hygiene water. The BDAT design and operation was previously described by Larsen et al. (2015); and the BAMBi was previously described by Kunzle et al. (2015). The recreated influent contained 12.5 g feces, 25 mL urine, and 2.5 g hand soap (Consumerline Products, Kenya) in 1 L of tap water. This influent mixture was prepared every 3-4 days, refrigerated at 4°C and stirred at ~50 RPM. The feces and urine were collected from a urine-diverting dry toilet at Eawag. Feces were collected weekly, homogenized with a blender, and stored at —20°C for up to 60 days. Urine was stored at room temperature for up to 48 h. In the BAMBi tank, the recreated influent is mixed with recirculated, treated water from the storage tank at a ratio of 1:20.

The full-scale BDAT treatment included a 50-L BAMBi tank with nine panels of polyethersulfone ultrafiltration membrane UP150 (Microdyn Nadir, Wiesbaden, Germany) with a nominal cut-off of 150 kDa, seeded with 0.5 L of municipal wastewater sludge. Aeration was achieved by pumping air (0.2-m^3^ h^—1^) through a perforated pipe underneath the membrane to maintain aerobic conditions on the exterior of the biofilm within the BAMBi tank. Water filtered through the BAMBI is then pumped into a 30-L clean water tank, which housed one or more of the post-treatment options: (1) GAC—to treat the remaining of organic matter and to reduce color (pore size of 0.6-1 mm, Norit Americas Inc., USA), (2) chlorination—to disinfect water using sodium hypochlorite (Fluka Chemie, Switzerland), and electrolysis—to provide on-site chlorine for disinfection (Condias, Germany).

### Collecting and Preparing Water Samples

Water samples (20 L) were collected from the clean water tanks of two BDATs: one operating with only the BAMBi (after BAMBi), and one with the addition of a 6 L-GAC column after the BAMBi (after BAMBi+GAC). The samples were prepared followed a method modified from Vital et al. (2008) to remove indigenous microorganisms. Indigenous microorganisms were removed to preserve water quality at the time of collection so that a consistent source water could be used for all studies. Otherwise, storage of water with indigenous community intact would have led to water quality changes due to continued microbial activity and may have interfered with growth of target bacteria. Specifically, samples were filtered through a 1-μm pressurized filter (Geberit, Germany), pasteurized at 80°C for 60 min, and filtered again through a 0.2-μm membrane filter (Whatman, UK).

Notably, pretreatment did not substantially affect the concentration or characterization of DOC, as shown from chromatograms measured using an LC-OCD instrument for water samples before and after the procedure of filtration and pasteurization [Figure S1, Supporting Information (SI)]. All samples were processed within 6 h after collection and stored at 4^0^C until used. In this study, prepared water is referred to water after mentioned pasteurization and filtration steps. Characteristics of prepared water samples, including concentration of anions $\left({\text{NO}}_{\text{2}}^{\text{-}}\text{, }{\text{NO}}_{\text{3}}^{\text{-}}\text{, }{\text{PO}}_{\text{4}}^{\text{3-}}\text{, }{\text{SO}}_{\text{4}}^{\text{2-}}\text{, }{\text{Cl}}^{\text{-}}\right)$ and cations (Ca^2^+, Mg^2^+, Na+, K+, Fe^3+/2+^, Mn^2^+, Cu^2^+, Ba^2^+), were analyzed by the Engineering Analytical Laboratory at Eawag.

### Bacterial Strains

Four bacteria used in this study include two isolated indicators from wastewater [*Escherichia coli* (GenBank KU737538) and *Enterococcus faecalis* (GenBank KU737539)] and two pathogens [*Pseudomonas aeruginosa* PAO1 (ATCC 15692) and *Salmonella typhimurium* SB300 (provided by Dr. M. Suar from Institute of Microbiology, ETH Hönggerberg, Switzerland)]. The wastewater isolates were confirmed as *E. coli* and *Ent. faecalis* using 16sRNA sequencing (Microsynth, Switzerland). All experiments involved the four bacteria of interest were conducted under proper procedures in a Biosafety Level 2 laboratory at Eawag. Further details of sequencing and bacterial preparation are provided in the SI.

### Growth Potential Experiments

The experiments were set up following the pathogen growth potential assay of Vital et al. (2010). Target bacteria were grown overnight and seeded at an initial concentration of ~10^3^ cells mL^—1^ with 20 ml prepared water samples (20 mL) into 40 mL carbon-free glass vials. Vials were then incubated at 30°C in 72 h in the dark to reach stationary phase. Samples (1 mL) were taken at the beginning, end and intermittently throughout to measure bacterial concentration using both flow cytometry and culture. Sterilized distilled water was included as negative controls. AOC concentration was defined by the growth of the indigenous community: 1 μg AOC L^—1^ = 10^7^ cells L^—1^ (Hammes and Egli, 2005).

### Bacterial Competition Assays

To measure growth of bacteria in competition with the indigenous community, vials with prepared water samples and bacteria of interest were inoculated with raw water samples such that the indigenous community was seeded to a concentration of 10^3^ cells mL^-1^.

### Nutrient Limitation Assays

Nutrient limitations to growth for the bacteria were determined by supplementing with additional sources of carbon (C) [300 mg L^-1^ sodium acetate (CH_3_COONa, Fluka Chemie, Switzerland)], nitrogen (N) [5 mg L^-1^ ammonium sulfate ((NH_4_)_2_SO_4_, Sigma- Aldrich, Germany)], phosphorus (P) [80mg L^-1^ sodium phosphate dibasic (Na_2_HPO_4_, Sigma-Aldrich, USA)], and iron (Fe) [5 mg L^-1^ ferric chloride (FeCl_3_, Sigma-Aldrich, USA)]. The concentrations were chosen to be at least 10 times higher than in concentrations of C, N, P, and Fe measured in the water after BAMBi.

Nutrient limitations for Ent. faecalis were further evaluated by supplementation of minimal media Davis broth (7g L^-1^ of K_2_HPO_4_, 2g L^-1^ of KH_2_PO_4_, 0.5 g L^-1^ of sodium citrate, 0.1 g L^-1^ of MgSO_4_, 1g L^-1^ of NH_4_SO_4_, supplemented with 100mgL^-1^ ofthiamine,0.1%glucose),vitamins [biotin,calcium, pantothenic acid, and pyridoxine (each at 20 μg mL^-1^), nicotinic acid and riboflavin (each at 2 μg mL^-1^), folic acid (0.2 μg mL^-1^)], and 20 amino acids (each at 20 μg mL^-1^) (Murray et al., 1993). All experiments were conducted in triplicates.

### Inactivation and Regrowth Experiments

#### Inactivation Experiments

Water after BAMBi+GAC was used for the inactivation and regrowth experiments. *E. coli* and P. aeruginosa were tested in the inactivation experiments due to their ability to grow in water after BAMBi+GAC without the need for additional nutrients (See Results section Growth of Bacteria in Waters from the BDAT). Overnight incubated cells were added to prepared water samples to reach final concentration of ~10^5^ cells mL^-1^, and then exposed to a disinfectant (chlorine or electrolysis). When chlorine was used directly as disinfectant, sodium hypochlorite (10%, Fluka Chemie, Switzerland) was added to the mixture of 100 mL of water after BAMBi+GAC to establish initial concentrations of 0.07, 0.14, 0.2, 0.5, 1.7, and 3.4 mg Cl_2_ mL^-1^. The initial concentrations of chlorine were 1x, 2x, 3x, 7x, 24x and 49x higher than the total chlorine demand of the water after BAMBi+GAC. The polyvinylidene fluoride electrolysis unit (Condias, Germany) used to produce disinfectants has a dimension of 4.5 x 3.5 x 22.5 cm (l x w x h) and four niobium substrate electrodes, each with a boron- doped diamond coating. The electrolysis unit was run at 0.5, 5, and 20 W at a recirculating flow rate of7 L h^-1^ in total of30 min. Samples were collected at 0, 5, 15, and 30 min after disinfection exposure. Free and total chlorine concentrations were measured using the DBD method (Hach LCK 310, Germany) at the same time as bacterial concentrations (Table S1). After each time point, sodium thiosulfate solution (Na_2_S_2_O_3_, stock concentration of 46 g L^-1^) was added to samples to quench residual chlorine prior to bacterial quantification. All experiments were conducted in triplicates.

#### Reactivation/Regrowth Experiments

To determine whether bacteria can reactivate or regrow after inactivation, water samples after 30 min of each inactivation experiment were aliquoted in 40 mL carbon free glass vials and incubated at 30°C in the dark for 72 h. A subset of the samples exposed to direct chlorination and electrolysis were treated with sodium thiosulfate solution (46 g L^-1^) to quench residual chlorine before regrowth experiments to isolate the impact of treatment from residual chlorine on reactivation or regrowth. An increase in bacterial concentration of more than 10 cell mL^-1^ after 72 h of incubation was considered as reactivation or regrowth. Additionally, to determine the effect of each posttreatment on bacterial growth potential, overnight incubated bacterial cells were also added to water samples after each inactivation experiment (initial concentration of 10^3^ cells mL^-1^). All experiments were conducted in triplicates.

### Bacterial Concentration Measurements

Culture and flow cytometry (FCM) methods were used to measure concentrations of bacteria in water samples. Samples were analyzed immediately after collection. Further details are in SI.

### Calculating Net Growth/Growth Potential and Growth Coefficient

#### Net Growth/Growth Potential

Net growth of bacteria or growth potential of a water sample is defined here as the difference between the final cell concentration in the stationary phase and the initial cell concentration at the beginning when the bacterial inoculum was introduced in the water sample (Vital et al., 2010). Bacteria were considered to grow in a water sample only when the minimum net growth was 10^3^ cells mL^-1^ (Vital et al., 2010).

#### Growth Coefficient

**FIGURE 1:**
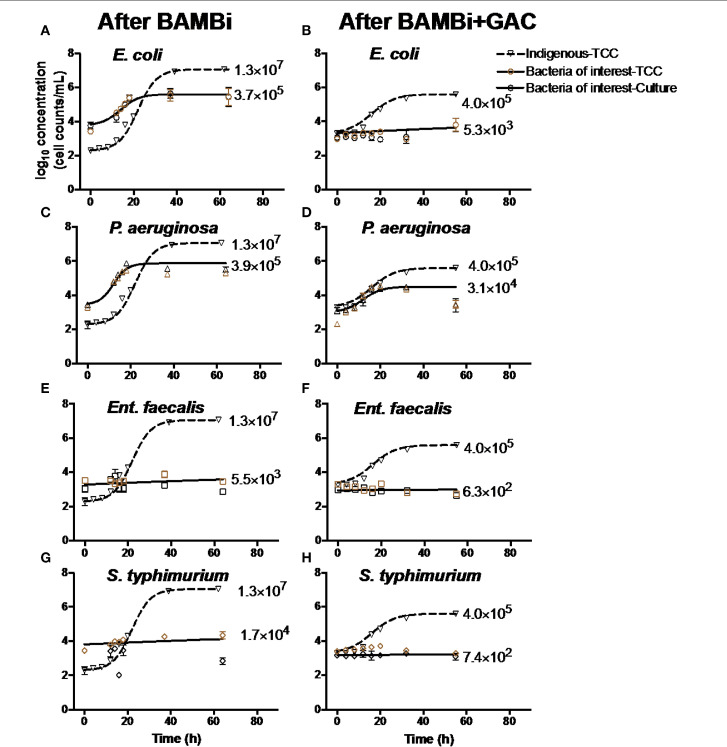
Growth curves of indigenous communities, (**A,B**) *E. coli*, (**C,D**) *P aeruginosa*, (**E,F**) *Ent. faecalis*, and (**G,H**) *S. typhimurium* in water after BAMBi and after BAMBi+GAC. Concentrations of bacteria were measured using culture method and FCM for TCC. Growth curves of the indigenous bacteria (▽) were added in each figure to compare with the growth of the bacteria of interest (o for TCC, and o for culture method). Negative controls and samples with *Ent. faecalis* and *S. typhimurium* did not show significant change in bacterial concentration during the course of the experiments. Net growth of each bacteria (CFU/mL) is shown next to each growth curve. More details of the growth curves can be found in Table S1.

A sigmoid function was used to fit an S-shaped growth curve to each growth potential data set shown in Figure 1 (Zwietering et al., 1990). Parameters of the sigmoid function for each data set are shown in Table S1.

$$y\;=\;y_{\min}\;+\;\frac{\left(y_{\max}\;-\;y_{\min}\right)}{1+10^{\left(\log EC50-t\right)\;\times \;r_{j}^{i}}}$$

where *y*_min_ and *y*_max_ are minimum and maximum values of log concentration, respectively. t is time (h), and logEC50 is *t*-value when *y*-value is halfway between *y*_min_ and *y*_max_. $r_{j}^{i}$ is the slope of the exponential phase, or growth coefficient of bacterium i (i = E for *E. coli*, P for *P. aeruginosa*, S for *S. typhimurium*, Ent for *Ent. faecalis*) in water sample j (j = BAMBi for water after BAMBi, and GAC for water after BAMBi+GAC).

#### Statistical Analysis

GraphPad Prism 6.0.1 (GraphPad Software, USA) was used to perform statistical tests. Non-linear regression was performed to fit the sigmoid function to each bacterial growth curve and calculate growth coefficients. Comparison of net growth, growth coefficients, and water characteristics was conducted using either paired *t*-tests or two-way ANOVA. All statistical tests assumed an alpha of0.05.

## RESULTS

### Water Characteristics

Water treated with the BAMBi had generally higher nutrient and metal ion concentrations than water treated with the BAMBi and GAC (Table 1). Specifically, DOC, ${\text{PO}}_{\text{4}}^{\text{3-}}$, ${\text{SO}}_{\text{4}}^{\text{2-}}$, and Cu^2+^ were statistically significantly lower in the BAMBi+GAC than the BAMBi (Student’s *t*-test with Sidak correction, p < 0.05, Table 1). Although reductions were observed for pH, AOC, ${\text{NO}}_{\text{3}}^{\text{-}}$, Cl^-^, Na^+^, K^+^, Ca^2+^, and Mg^2+^, the reduction was not statistically significant (Student’s *t*-test with Sidak correction, *p* > 0.05).

**TABLE 1:** Characteristics of water after BAMBi and after BAMBi+GAC.

Parameters	After BAMBi	After BAMBi+GAC	*p*-value (Student’s *t*-test)	**Adj. *p*-value (Sidak correction)**
PH	8.3 ± 0.0	7.9 ± 0.1	8 x 10^-3^	0.09
DOC (mg L^-1^)	36.5 ± 1.3	7.9 ± 0.1	2.5 x 10^-8^	3 x 10^-7^
AOC (mg L^-1^)	0.3 ± 0.1	0.1 ± 0.1	0.53	1.00
NO^-^-N (mg L^-1^)	<2	<2	NA	NA
NO^-^-N (mg L^-1^)	25 ± 1.0	21.1 ± 1.1	0.07	0.60
PO|^-^-P (mg L^-1^)	16.5 ± 1.8	11.5 ± 1.2	4.3 x 10^-4^	5 x 10^-3^
So2^-^-S (mg L^-1^)	74.5 ± 2.9	100.7 ± 6.7	1.4 x 10^-5^	1.7 x 10^-4^
Cl^-^ (mg L^-1^)	133.8 ± 2.5	126.3 ± 2.4	0.022	0.24
NH+-N (mg L^-1^)	<0.2	<0.2	NA	NA
Ca^2^+ (mg L^-1^)	2.5 ± 0.1	2.3 ± 0.1	0.21	0.94
Mg^2^+ (mg L^-1^)	3.7 ± 0.1	3.6 ± 0.2	0.81	1.00
Na+ (mg L^-1^)	78.4 ± 0.1	71.9 ± 4.9	0.22	0.95
K+ (mg L^-1^)	100.5 ± 1.8	96.7 ± 6.3	0.57	1.00
Fe^3^+^/2^+ (μg L^-1^)	<5	<5	NA	NA
Mn^2^+ (μg L^-1^)	<5	<5	NA	NA
Cu^2^+ (μg L^-1^)	11.8 ± 0.7	<5	8.5 x 10^-6^	1 x 10^-4^
Ba^2^+ (μg L^-1^)	<5	<5	NA	NA

*AOC concentration was determined from the growth of indigenous bacteria. Data is shown as mean *±* standard error. Sample size per measurement is 6. Student’s t-test was used to determine significant differences between BAMBi and BAMBi+GAC, as shown with p-value and adj. p-value, where adj . p-value is Sidak correction forfamilywise errorrates. NA refers to Not Applicable, and is used when samples were below the lower limit of detection*.

### Growth of Bacteria in Waters from the BDAT

The indigenous microbial community, E. coli, and P. aeruginosa, grew in both waters during storage, whereas Ent. faecalis and S. typhimurium only grew in water after treated with BAMBi (Figure 1). The indigenous microbial community grew to a higher concentration in water after treatment with the BAMBi alone (Net growthBAMBi = 1.3 x 107 cells mL-1) as compared to the BAMBi+GAC (4.0 x 105 cells mL-1, Figure 1). The indigenous community also grew faster in water after BAMBi (r*BAMBi* = 0.1 h-1) than BAMBI+GAC (*r_gac_* = 0.08 h-1, Table S1). Similarly, the growth of both P. *aeruginosa* and *E. coli* in water treatment with BAMBi was higher than in water treated with BAMBi+GAC (56% more for *P. aeruginosa*, and 38% more for *E. coli*, Table S1). While P. *aeruginosa* had similar growth coefficient in both waters (Table S1), *E. coli* grew faster in water after BAMBi compared to in water after BAMBi+GAC $\left(r_{BAMBi}^{E.\;coli}\;=\;10r_{GAC}^{E.\;coli}\right)$.

There was a good agreement between TCC data and culture data for all four bacteria of interest in both water samples (twoway ANOVA, *p* > 0.05).

### Factors Influencing Bacterial Growth

#### Nutrient Addition

**FIGURE 2:**
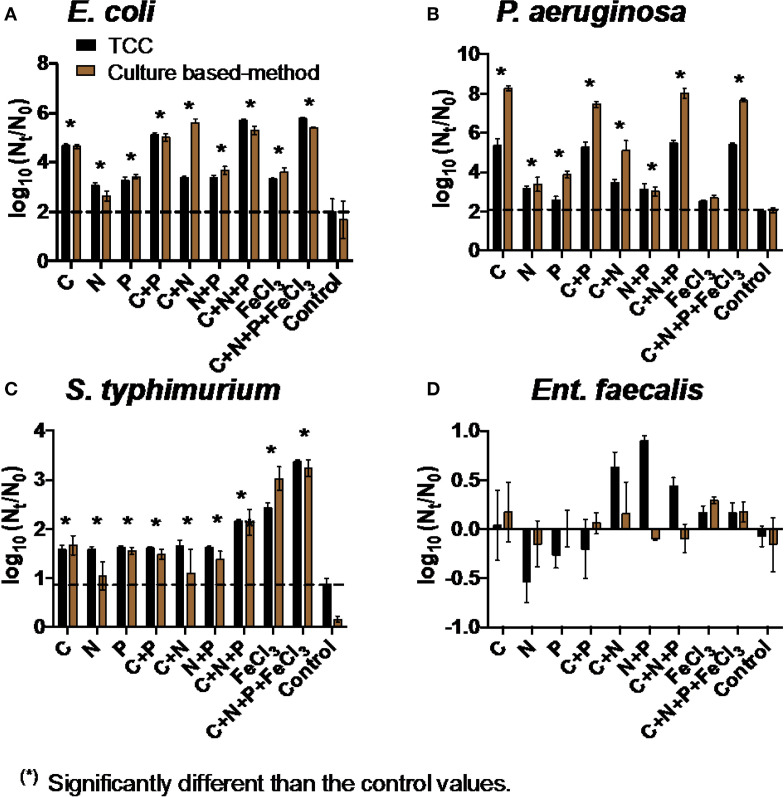
Net growth [log(N^t^/N^O^)] of **(A)** *E. coli*, **(B)** *P aeruginosa*, **(C)** *S. typhimurium*, and **(D)** *Ent. faecalis* in the addition of C, N, P, and Fe in water after BAMBi after 72 h. Controls refer to water after BAMBi without any nutrient supplementation. Dashed lines were drawn for comparison with the highest value of the controls in each experiment. Star symbols indicated that the values were significantly higher than the corresponding control values according to two-way ANOVA test for both TCC and culture data (p < 0.05). No significant differences were observed for *Ent. faecalis*.

The addition of all four nutrients (C, N, P, and Fe) individually or in combination increased the net growth of *E. coli* and *S. typhimurium* compared to the control samples (two-way ANOVA, p < 0.05, Figure 2). The growth of *P. aeruginosa* significantly increased when C, N, and P were added (two-way ANOVA, p < 0.05, Figure 2). The addition of C appeared to dramatically increase the growth of *E. coli* and *P. aeruginosa* (approximately two times higher compared to the addition of other nutrients). The growth of *S. typhimurium* approximately doubled with the addition of Fe compared to other nutrients.

Although the addition of C, N, P, and Fe did not show any positive effect on the growth of Ent. faecalis in water after BAMBi (Figure 2D), at least some bacterial growth was observed in the presence of minimal media (Davis media), amino acids, or vitamins with the largest effect size observed in the presence of all three (Figure S3).

There was a good agreement between TCC data and culture data for *E. coli, Ent. faecalis*, and *S. typhimurium* (two-way ANOVA, p > 0.05).

#### Indigenous Communities

**FIGURE 3:**
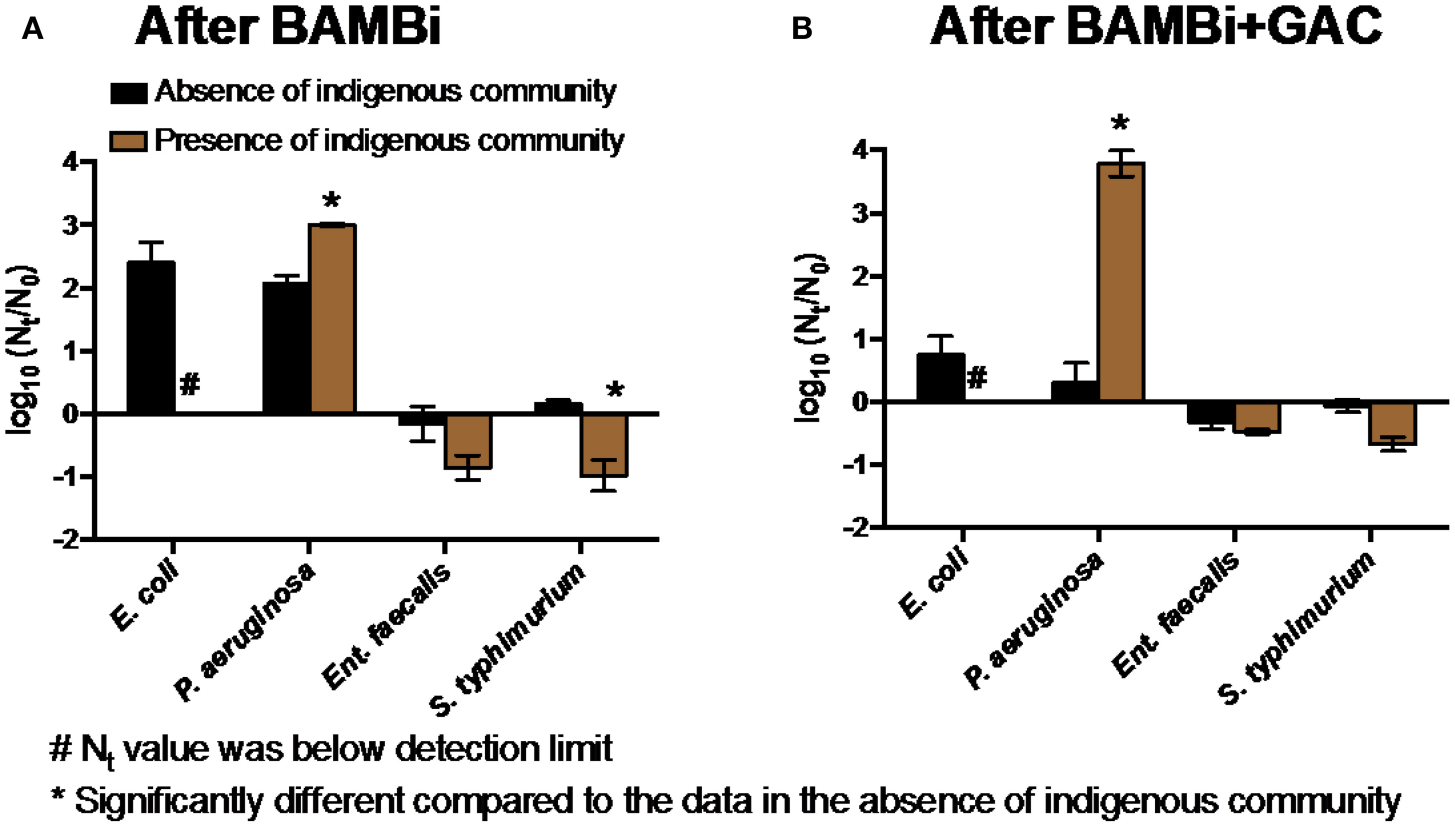
Net growth [log_10_(*N_t_/N_0_*)] of four tested bacteria in the presence and absence of **(A)** the BAMBi indigenous community in water after BAMBi and **(B)** the GAC indigenous community in water after BAMBi+GAC. Concentration at *t* = 72 h of *E. coli* in the presence of the indigenous communities was below detection limit (*N_t_* < 10 CFU mL-1). Star symbols indicated that the values in the presence and absence of the indigenous communities were significantly different (two-way ANOVA test, *p* < 0.05).

The presence of an indigenous microbial community typically, but not always, reduced the extent of growth of the target bacteria *E. coli, Ent. faecalis*, and *S. typhimurium*, but supported the growth of *P. aeruginosa* (Figure 3). Inhibition of *E. coli* appeared greatest: *E. coli* was not detected (Nt < 10 cell mL-1) after 72 h of incubation with the indigenous communities in both waters despite growth in the absence of the indigenous community. The indigenous community increased the growth of *P. aeruginosa* (two-way ANOVA, p < 0.05), with the BAMBi+GAC community having a bigger effect on the bacterial growth than the BAMBi community (Figure 3).

#### Disinfection Processes

##### Inactivation

**FIGURE 4:**
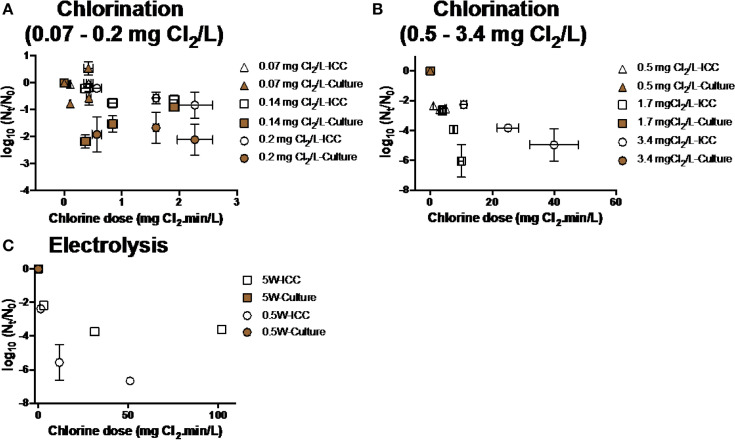
Inactivation of *E. coli* at various conditions during **(A,B)** chlorination and **(C)** electrolysis. Note that samples were measured using both FCM and culture based method data at every single time point. Culture data of some of the time points was not shown because their Nt-values were below detection limit (<10 CFU mL^-1^).

**FIGURE 5:**
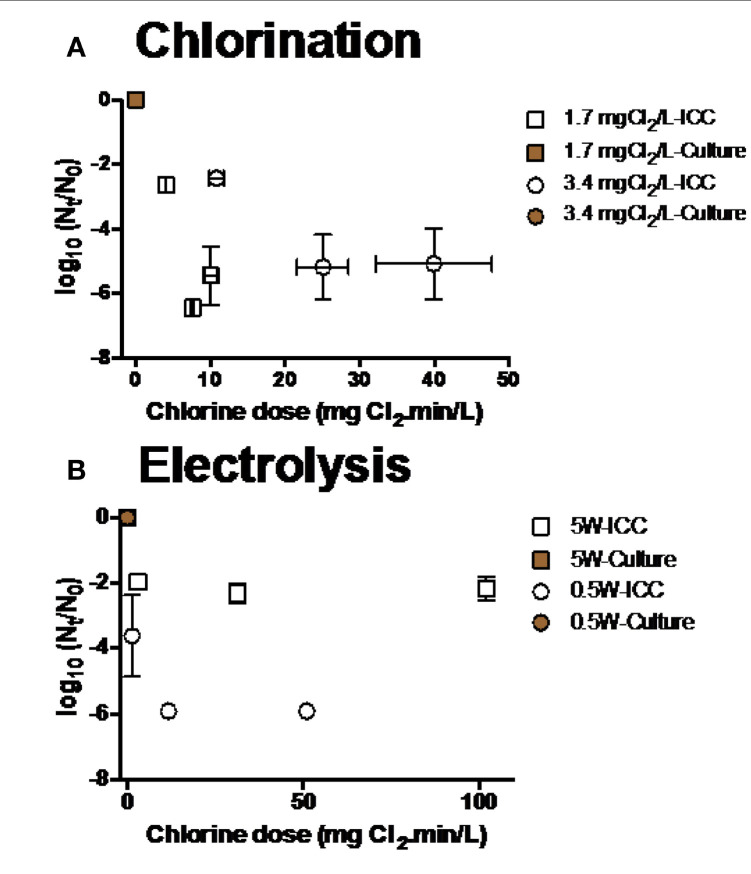
Inactivation of *P. aeruginosa* at various conditions during **(A)** chlorination and **(B)** electrolysis. Culture data of time points (*n* = 6) was not shown because their *N*_t_-values were below detection limit (<10 CFU mL−1).

Chlorination and electrolysis treatment options were tested for the efficiency of inactivation of *E. coli* and *P. aeruginosa*. Inactivation of *E. coli* was measured at various initial chlorine concentrations (0.07-3.4 mg Cl_2_ L^-1^, **Figures 4A****,B**). As chlorine demand of water after BAMBi+GAC was 0.14 mg Cl_2_ L^-1^, there was no observed inactivation of *E. coli* when chlorine concentrations were lower or equal to 0.14mg Cl_2_ mL^-1^. At higher initial chlorine concentrations (>0.5mg Cl_2_ L^-1^), more than 5 log_10_ inactivation of culturable *E. coli* was achieved after 5 min. Electrolysis at different intensities (0.5-20 W) was also shown to be effective in inactivating *E. coli* (>5 log_10_ inactivation after 5 min at all tested intensities of electrolysis, **Figure 4C**). Chlorination and electrolysis were shown to be effective in inactivating *P. aeruginosa* (>5 log_10_ inactivation after 5 min, **Figures 5A****,B**). Concentration of free and total chlorine during each electrolysis and chlorination experiment is shown in Figure S2 and Table S1, respectively.

There was a significant discrepancy between results from culture and FCM methods for every disinfection treatment: Viable cells shown by culture data were significantly lower than ICC data (two-way ANOVA, *p* < 0.05).

##### Reactivation/Regrowth of bacteria after disinfection

**FIGURE 6:**
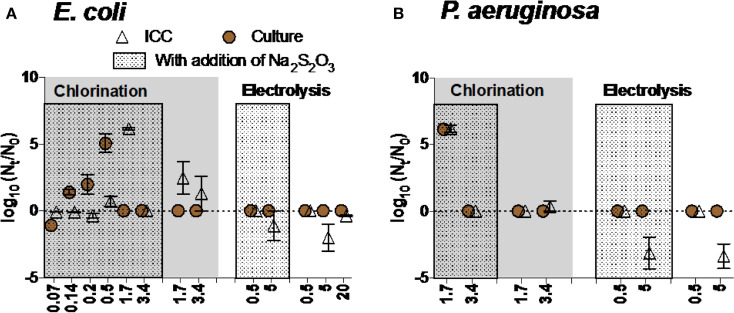
Reactivation and/or regrowth of **(A)** *E. coli* and **(B)** *P. aeruginosa* after inactivation experiments (chlorination and electrolysis). Data within boxes were from experiments in which Na2S2O3 was added to quench residual chlorine. Note that no bacteria were added after disinfection and before reactivation/regrowth experiments. Negative values reflect continued inactivation or loss of bacteria during incubation time.

Different disinfection methods variably influenced the likelihood of reactivation and/or regrowth of bacteria. For chlorination, E. coli inactivated when exposed to low concentrations of chlorine (from 0.14 to 0.5 mg Cl_2_ L^-1^) reactivated in the presence of Na_2_S_2_O_3_-a residual chlorine quencher (**Figure 6A**). No reactivation/regrowth of E. coli was observed after exposure to higher concentration of chlorine (3.4 mg Cl_2_ L^-1^) or electrolysis at various intensities, regardless of adding Na_2_S_2_O_3_ (**Figure 6A**). We observed increases in intact cell count—but not culturable cell count—in water exposed to 1.7 mg Cl_2_ L^-1^. This result—an outlier—is inconsistent with the rest of the data, and though we can speculate on the cause (i.e., flow cytometry error, incorrect gating by flow cytometer) we do not have evidence to support these speculations.

A similar trend was observed for P. aeruginosa: reactivation occurred after exposure to low concentration of chlorine in the presence of Na_2_S_2_O_3_ (1.7 mg Cl_2_ L^-1^) (Figure 6B). Electrolysis seemed to be effective in inhibiting reactivation of P. aeruginosa even in the presence of Na_2_S_2_O_3_.

##### Growth of bacteria in disinfected water

**FIGURE 7:**
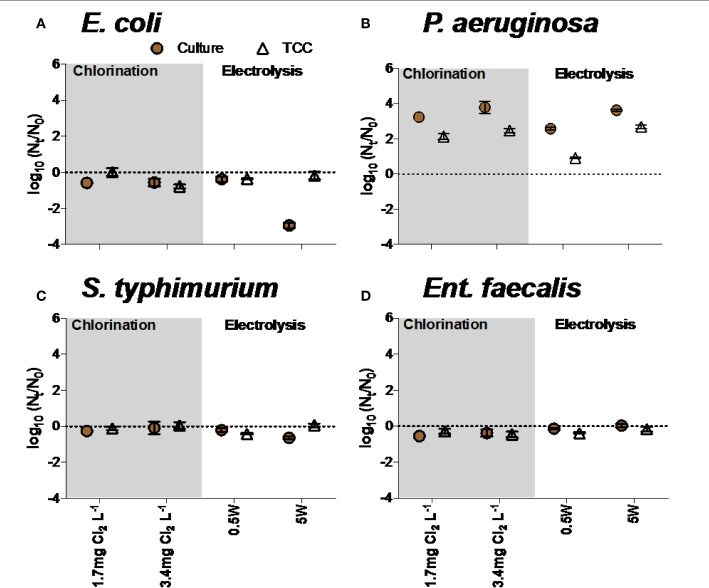
Growth of **(A)** *E. coli*, **(B)** *P. aeruginosa*, **(C)** *S. typhimurium*, and **(D)** *Ent. faecalis* in water after chlorination and electrolysis. Na2S2O3 was added in all experiments to quench residual chlorine. Note that negative values reflect continued inactivation or loss of bacteria during incubation time.

Bacterial growth was occasionally observed in disinfected water, influenced by bacterial species as well as level and type of treatment. For example, P. aeruginosa grew in waters after exposure to electrolysis and chlorine, but Ent. faecalis was not able to grow in any conditions (**Figures 7B****,D**). *E. coli* and *S. typhimurium* were unable to grow after chlorination and electrolysis treatments (**Figures 7A****,C**).

The TCC data and culture data in experiments with E. coli and P. aeruginosa were significantly different (two-way ANOVA, p < 0.05), but not in experiments with S. typhimurium and Ent. faecalis (two-way ANOVA, p > 0.05).

## DISCUSSION

Treatment of water for reuse is a promising strategy to increase water safety. However, hygienic risks increase during storage. Here, we demonstrate that bacterial species, water quality characteristics, and level and type of treatment all influence hygiene risks of water stored for reuse.

### Effects of Water Characteristics on Bacterial Growth

#### Nutrients

Nutrient availability strongly influenced bacterial growth in water stored for reuse. C was an important nutrient in our study. Growth of the indigenous community (which was used to indicate AOC concentration) decreased following water treatment with GAC (**Table 1**). The growth of *E. coli* and *P. aeruginosa* increased dramatically with the addition of C, indicating that C was the main limiting factor for bacterial growth (Morita, 1993; Vital et al., 2008). The maximum concentrations of *E. coli* and *P. aeruginosa* were always lower than the indigenous communities (**Figure 1**). This result aligns with Vital et al. (2008) reporting that the source, type and composition of C has strong effects on bacterial growth. In relation to the water treatment system in the BDAT, this finding emphasizes the need for an efficient removal of DOC in the feed water. The combination of BAMBi and GAC reduces ~95% of total DOC concentration, which helps limit bacterial growth during storage.

For *Ent. faecalis*, C (in the form of acetate), N, P, or Fe did not improve growth. Addition of Davis media, vitamins, or amino acids were required for growth, implying growth was limited by lack of specific nutrients, not by inhibitory substances. For *S. typhimurium*, the limiting nutrient was Fe, confirming findings of a previous study showing increased growth of *S. typhimurium* and other enteric pathogens in Fe-supplemented water (Kortman et al., 2012). Within the BDAT, blood (i.e., menstrual blood) may introduce iron into the system. The impacts of the introduction of blood as a source of iron should be monitored, as our results suggest *S. typhimurium* (and potentially other pathogens) is limited by Fe.

Our study also highlighted that growth in water stored for reuse depends on the characteristics of the bacteria. Only *E. coli* and *P. aeruginosa* were able to grow in water samples following treatment with BAMBi and GAC. *P. aeruginosa* had a faster growth coefficient and higher final concentration than *E. coli*, indicating that *P. aeruginosa* was more ubiquitous and had a larger nutrient pool than E. coli. This result agrees with findings from previous studies showing that P. aeruginosa were able to grow relatively fast to reach high cell concentration in waters with limited nutrients (e.g., distilled water from hospitals, tap water; Favero et al., 1971; van der Kooij et al., 1982). Because P. aeruginosa is ubiquitous in the environment, it is likely that it will colonize the BDAT system during operation. Our results highlight that carbon control via the BAMBi and GAC treatments will not be sufficient: additional controls (i.e., electrolysis) are needed.

#### Indigenous Microbial Community

We demonstrated that the indigenous microbial community is generally—but not always—antagonistic to the growth of pathogens in the water (**Figure 3**). Previous studies showed the same antagonism (Chandran and Mohamed Hatha, 2005; Vital et al., 2012; Van Nevel et al., 2013). Antagonism may arise from nutrient (like C) competition, production of inhibitory compounds (e.g., antibiotics, bacteriocins), or predation and parasitism (e.g., bacteriophage, protozoa, invertebrates) (Hibbing et al., 2010; Vital et al., 2012; Wang et al., 2013). However, we also observed a protagonistic effect for *P. aeruginosa*, as also shown elsewhere for both *E. coli* and Klebsiella pneumonia (Moreira et al., 1994; Kerr et al., 1999). One potential explanation is that P. aeruginosa was able to use intermittent or end products that were synthesized by the indigenous community (Sherr and Sherr, 2002).

It should be noted that the water samples had been filtered and pasteurized to remove the original indigenous community to preserve the water quality during the course of the study (see section Collecting and Preparing Water Samples). In the BDAT system, the indigenous community is expected to be always present in the planktonic phase and/or in the biofilms. As a result, the interaction between the indigenous community and the pathogens (e.g., nutrient competition, production of inhibitory compounds, and predation) in the BDAT system likely differs from that observed. Additional research is needed to confirm dynamics observed in the laboratory align with dynamics observed in the BDAT under operating conditions (Ziemba et al., in preparation).

### Effects of Inactivation Processes on Reactivation and Growth of Bacteria

The reactivation/regrowth of *E. coli* and *P. aeruginosa* depended on the water and disinfectant characteristics (**Figure 6**). Bacterial reactivation occurred after exposed to low concentrations of chlorine without residual, as observed in previous studies (Jjemba et al., 2010; Li et al., 2013). *E. coli* and *P. aeruginosa* might enter the viable but non-culturable (VBNC) state under certain conditions during chlorination (Oliver, 2010), and therefore be able to reactivate.

No reactivation was observed after electrolysis, even in the presence of chlorine quencher. This result may be due, in part, to production of disinfectants (e.g., hydroxyl, nitrate radical, or phosphate radical besides chlorine that prevent formation of a reactivating VBNC state (Kerwick et al., 2005; Guitaya et al., 2015). We also suspect that the presence of reactive oxidants may change carbon structure and thereby remove available carbon required for regrowth.

As *P. aeruginosa* was shown to grow after being spiked into disinfected water without residual chlorine, pathogen contamination is potentially a high risk that can affect the system hygienic performance besides regrowth/reactivation. During storage, pathogen contamination can happen via multiple events, including malfunction of one or more treatment processes, access to storage tank by hosts that contain pathogens (e.g., aquatic organisms, insects, birds, rodents, contaminated human hands). Therefore, to ensure the safety of reusing treated fecal- contaminated water, it is important to manage the access of the storage tank carefully, maintain a cleaning procedure for the tank regularly, and have a proper residual of disinfectant constantly.

In this study, we investigated inactivation and reactivation/regrowth of *E. coli* and *P. aeruginosa* in the absence of an indigenous community. The indigenous microbiota were removed to prevent microbial degradation of source water during storage and to allow quantification of bacteria using culture-based methods and flow cytometry simultaneously. The indigenous community may influence inactivation and/or reactivation/regrowth rates ofbacteria. For example, in studies of chlorination of both reclaimed and drinking water, inactivation using chlorine has been shown to result in tailing of intact cell counts of the indigenous microbial community (Ramseier et al., 2011; Li et al., 2013). This phenomenon has been attributed to a resistant sub-population and/or shielding through bacterial aggregation or particle adsorption (Ramseier et al., 2011). However, Li et al. (2013) demonstrated inactivation of in situ total coliforms, enterococci, and*Salmonella* spp. in reclaimed water at rates similar to those observed here. We therefore expect similar pathogen inactivation and reactivation/regrowth kinetics in studies including the indigenous communities, but further research is warranted to conclusively demonstrate this.

### Comparison between Culture and FCM Methods

In general, conclusions from culture and FCM methods aligned for experiments measuring bacterial growth, but differed for inactivation experiments. The discrepancy between culture and FCM methods observed for inactivation was likely because ICC measures intact cell membranes for bacteria that may not be culturable. The ICC measurement relies on cell staining with Propidium Iodide which only stains cells with damaged membranes (Berney et al., 2007). Some dead or unculturable cells may have intact membranes, so ICC data is considered more conservative (i.e., overestimate concentrations of surviving cells) than culture (Joux and Lebaron, 2000; Bosshard et al., 2010).

### Implication for Water Reuse

Concerns related to pathogen growth in drinking water and reclaimed water have been raised in previous studies (van der Kooij, 2003; Oesterholt et al., 2007; Jjemba et al., 2010; Weinrich et al., 2010). In this study, we demonstrated that the growth of pathogens in water stored for reuse in an onsite sanitation technology (i.e., the BDAT) is a potential concern and, therefore, increases health risks. A bacterial indicator (*E. coli*) and an opportunistic pathogen (*P. aeruginosa*) were able to grow in water stored for reuse after multiple, different effective treatment processes. Water characteristics, including nutrient concentrations and indigenous bacterial communities, were shown to have strong effects on the bacterial growth. Additionally, we demonstrated the complex and nonuniform influence of indigenous communities on the growth of pathogens.

We also demonstrated that the choice of treatment processes was a key factor influencing bacterial growth. The presence of residual disinfectants (e.g., chlorine and/or other reactive active species) contributed to water biostability. Among three disinfection options, electrolysis appeared to be the most effective method to inhibit pathogen growth, followed by chlorination with high chlorine concentrations (e.g., >0.5 mg Cl_2_ L^-1^ for *E. coli* and >1.7 mg Cl_2_ L^-1^ for *P. aeruginosa*).

### Limitation of the Study

There were notable limitations to this study. First, we only tested growth, inactivation, and reactivation of four bacteria of interest. There is heterogeneity in both growth and inactivation coefficients of the four tested bacterial species, so future research is likely needed to investigate other pathogens (i.e., *Legionalla* spp.) to ensure safety of water reuse.

Second, the study focused on the growth and inactivation of bacteria in the planktonic phase. Biofilms in storage tanks, like the clean water tank where water is stored for reuse in the BDAT, likely also influence growth and inactivation of bacteria. As aggregates of microbial cells attach to surfaces, biofilm members have advantages of being protected from disinfectants and predators compared to planktonic cells (LeChevallier et al., 1988; Flemming and Wingender, 2010). In addition, the matrix of hydrated extracellular polymeric substances (EPS) helps biofilm members consume complex substrates (e.g., humic acids) as food, which are not bioavailable for planktonic bacteria (Fischer, 2003; Flemming and Wingender, 2010). More research is needed to understand the growth and inactivation of bacteria in biofilms in water stored for reuse, especially in the context of the BDAT.

Third, there is a discrepancy between conditions in the laboratory and in the real BDAT system. In our laboratory setup, all experiments were conducted in batch reactors, in which the concentration of nutrients, residual disinfectant, and bacteria drastically changes in the course of the experiments in 72 h. These conditions are different to those in the BDAT, where water after BAMBi is frequently added into the clean water tank and treated water is frequently removed upon every usage. The continuous flow conditions in the BDAT create a certain level of consistency in the bulk-phase concentration of nutrients and bacteria for prolonged periods of time. The concentration of residual disinfectant in the clean water tank of the BDAT is also kept constant with a regular dosing interval. Another difference between laboratory setup and real-life conditions is that the indigenous community is constantly present in the BDAT system whereas it was removed from the water samples for the subset of laboratory experiments on inactivation and reactivation/regrowth. The indigenous community in the BDAT may impact the inactivation and reactivation/regrowth of pathogens, for example by shielding and/or providing. Results learnt from inactivation experiments, for example the disinfectant demand for a sufficient pathogen removal, likely need to be adapted in the presence of the indigenous community in the BDAT conditions.

## CONCLUSIONS

Reuse of treated fecally-contaminated water is a promising strategy to safely increase water efficiency. However, water must be sufficiently treated to reduce hygiene risks associated with reuse. Storage and distribution of water for reuse, in particular, pose potential health risks. We demonstrated these risks through the following observations:

- Both *E. coli* and opportunistic pathogen *P. aeruginosa* grow in water following treatment unless there is sufficient disinfectant residual.
- Growth during storage is influenced by both water quality and bacterial species: although neither *Ent. faecalis* nor *S. typhimurium* grew in treated water, the addition of limiting nutrients was able to initiate growth.
- GAC combined with chlorination (sufficiently high concentrations, with residual) or electrolysis is effective additional treatment that both reduce microorganisms in the water and limits regrowth potential.

## SUPPLEMENTARY MATERIAL

The Supplementary Material for this article can be found online at: https://www.frontiersin.org/articles/10.3389/fenvs. 2017.00090/full#supplementary-material
