# Supplementary material for: Controlling Bacterial Pathogens in Water for Reuse: Treatment Technologies for Water Recirculation in the Blue Diversion Autarky Toilet
Source: Front Environ Sci. 2017 Dec 19;5:90. doi: 10.3389/fenvs.2017.00090 (PMC7705130; doi:10.3389/fenvs.2017.00090)
Supplement: Supplementary file 1 [file FES-05-090-s001.docx]

**Controlling Bacterial Pathogens in Water for Reuse: Treatment Technologies for Water Recirculation in the Blue Diversion Autarky Toilet**

Mi T. Nguyen^1,2^, Lukas Allemann^1^, Christopher Ziemba^1,3^, Odile Larive^1,4^, Eberhard Morgenroth^1,3^, Timothy R. Julian^1^

^1^ Eawag, Swiss Federal Institute of Aquatic Science and Technology, 8600 Dübendorf, Switzerland

^2^ Nguyen Tat Thanh University, Ho Chi Minh City, Vietnam

^3^ETH Zürich, Institute of Environmental Engineering, 8093 Zürich, Switzerland

^4^ EPFL, School of Architecture, Civil and Environmental Engineering, 1015 Lausanne, Switzerland

**Bacteria preparation for experiments**

The bacteria were stored as glycerol stocks at -80°C. The frozen stocks were streaked onto lysogeny broth (LB) agar plates (Sigma-Aldrich, USA) and incubated at 37°C for 24 h. Cultures were prepared fresh daily by inoculating one colony into brain heart infusion broth (Sigma-Aldrich, India) for *Ent. faecalis* or LB broth (Sigma-Aldrich, USA) for the three other bacteria. Cultures were then incubated at 37°C for 24 h to reach stationary phase. Cells were pelleted by centrifuging at 6,800×g for 3 mins, then washed and resuspended with autoclaved nanopure water (Barnstead Nanopure, Switzerland). Bacterial cells were then added into water samples to reach appropriate initial concentration of each experiment.

**Bacterial concentration measurements**

*Culture.* Spread plating aliquots of 20 μl on selective media corresponding to each bacteria (Table 2) was used to enumerate culturable bacterial. Selective media used for culturing *E. coli*, *P. aeruginosa*, *S. typhimurium*, and *Ent. faecalis* were mTEC agar (Sigma-Aldrich, India), GSP agar (Sigma-Aldrich, India), Leifson agar (Sigma-Aldrich, Spain), and mEnterococcus agar (Sigma-Aldrich, Switzerland), respectively. Analysis for each sample was conducted in triplicate.

*Flow cytometry.* FCM was used to determine concentration of total bacterial cells (total cell counts, TCC), or intact bacterial cells (intact cell counts, ICC) (Berney et al., 2008; Hammes et al., 2008). SYBR Green I stain (Molecular Probes, Switzerland) diluted 100 times in dimethyl sulfoxide (DMSO, Fluka Chemie, Switzerland) was used as dye for TCC analysis. To measure concentration of damaged cells in the inactivation-regrowth experiments, propidium iodide (PI, Molecular Probes, Switzerland, 30 mM) was mixed with SYBR Green I stock at a ratio of 1:50 was used to measure ICC. Samples were incubated with dye in 10 min at 37°C in the dark before analysis. All samples were measured on a BD Accuri C6 (BD, USA) equipped with a solid-state laser excitation at a fixed wavelength of 488 nm. The trigger was set on the green fluorescence channel at 530 nm, and signals for cell counts were collected on a combined 530 nm/670 nm dot plots. Samples were diluted before analysis if it contained more than 10^6^ cells mL^-1^.

**16S rRNA sequences of *E. coli* and *Ent. faecalis* isolates**

*E. coli* isolate sequence – GenBank accession No. KU737538

GCTTGCTKCTTYGCTGACGAGTGGCGGACGGGTGAGTAATGTCTGGGAAACTGCCTGATGGAGGGGGATAACTACTGGAAACGGTAGCTAATACCGCATAACGTCGCAAGACCAAAGAGGGGGACCTTCGGGCCTCTTGCCATCGGATGTGCCCAGATGGGATTAGCTAGTAGGTGGGGTAACGGCTCACCTAGGCGACGATCCCTAGCTGGTCTGAGAGGATGACCAGCCACACTGGAACTGAGACACGGTCCAGACTCCTACGGGAGGCAGCAGTGGGGAATATTGCACAATGGGCGCAAGCCTGATGCAGCCATGCCGCGTGTATGAAGAAGGCCTTCGGGTTGTAAAGTACTTTCAGCGGGGAGGAAGGGAGTAAAGTTAATACCTTTGCTCATTGACGTTACCCGCAGAAGAAGCACCGGCTAACTCCGTGCCAGCAGCCGCGGTAATACGGAGGGTGCAAGCGTTAATCGGAATTACTGGGCGTAAAGCGCACGCAGGCGGTTTGTTAAGTCAGATGTGAAATCCCCGGGCTCAACCTGGGAACTGCATCTGATACTGGCAAGCTTGAGTCTCGTAGAGGGGGGTAGAATTCCAGGTGTAGCGGTGAAATGCGTAGAGATCTGGAGGAATACCGGTGGCGAAGGCGGCCCCCTGGACGAAGACTGACGCTCAGGTGCGAAAGCGTGGGGAGCAAACAGGATTAGATACCCTGGTAGTCCACGCCGTAAACGATGTCGACTTGGAGGTTGTGCCCTTGAGGCGTGGCTTCCGGAGCTAACGCGTTAAGTCGACCGCCTGGGGAGTACGGCCGCAAGGTTAAAACTCAAATGAATTGACGGGGGCCCGCACAAGCGGTGGAGCATGTGGTTTAATTCGATGCAACGCGAAGAACCTTACCTGGTCTTGACATCCACGGAAGTTTTCAGAGATGAGAATGTGCCTTCGGGAACCGTGAGACAGGTGCTGCATGGCTGTCGTCAGCTCGTGTTGTGAAATGTTGGGTTAAGTCCCGCAACGAGCGCAACCCTTATCCTTTGTTGCCAGCGGTCCGGCCGGGAACTCAAAGGARACTGCCAGTGATAAACTGGAGGAAGGKGGGGATGACGTCAAGTCATCATGGCCCTTACGACCAGGGCTACCMM

*Ent. faecalis* isolate sequence – GenBank accession No. KU737539

CGNNCGCTTCTTTCCTCCCGAGTGCTTGCACTCAATTGGAAAGAGGAGTGGCGGACGGGTGAGTAACACGTGGGTAACCTACCCATCAGAGGGGGATAACACTTGGAAACAGGTGCTAATACCGCATAACAGTTTATGCCGCATGGCATAAGAGTGAAAGGCGCTTTCGGGTGTCGCTGATGGATGGACCCGCGGTGCATTAGCTAGTTGGTGAGGTAACGGCTCACCAAGGCCACGATGCATAGCCGACCTGAGAGGGTGATCGGCCACACTGGGACTGAGACACGGCCCAGACTCCTACGGGAGGCAGCAGTAGGGAATCTTCGGCAATGGACGAAAGTCTGACCGAGCAACGCCGCGTGAGTGAAGAAGGTTTTCGGATCGTAAAACTCTGTTGTTAGAGAAGAACAAGGACGTTAGTAACTGAACGTCCCCTGACGGTATCTAACCAGAAAGCCACGGCTAACTACGTGCCAGCAGCCGCGGTAATACGTAGGTGGCAAGCGTTGTCCGGATTTATTGGGCGTAAAGCGAGCGCAGGCGGTTTCTTAAGTCTGATGTGAAAGCCCCCGGCTCAACCGGGGAGGGTCATTGGAAACTGGGAGACTTGAGTGCAGAAGAGGAGAGTGGAATTCCATGTGTAGCGGTGAAATGCGTAGATATATGGAGGAACACCAGTGGCGAAGGCGGCTCTCTGGTCTGTAACTGACGCTGAGGCTCGAAAGCGTGGGGAGCAAACAGGATTAGATACCCTGGTAGTCCACGCCGTAAACGATGAGTGCTAAGTGTTGGAGGGTTTCCGCCCTTCAGTGCTGCAGCAAACGCATTAAGCACTCCGCCTGGGGAGTACGACCGCAAGGTTGAAACTCAAAGGAATTGACGGGGGCCCGCACAAGCGGTGGAGCATGTGGTTTAATTCGAAGCAACGCGAAGAACCTTACCAGGTCTTGACATCCTTTGACCACTCTAGAGATAGAGCTTTCCCTTCGGGGGCAAAGTGACAGGTGGTGCATGGTTGTCGTCAGCTCGTGTCGTGARATGTTGGGTTAAGTCCCGCAACGAGCGCAACCCTTATTGTTAGTTGCCATCATTTAGTTGGGCMCTCTAGC

**LC-OCD measurement**

LC-OCD sampling and testing was conducted with glass vials muffled at 450˚C for 12 hours. Separation was achieved with a Toyopearl TSK HW-50S column (Tosoh Bioscience, King of Prussia, PA, USA) with a range of 100–20,000 Da and carbon was quantified using an infrared detector (DOC-Labor Dr. Huber, Karlsruhe, Germany).


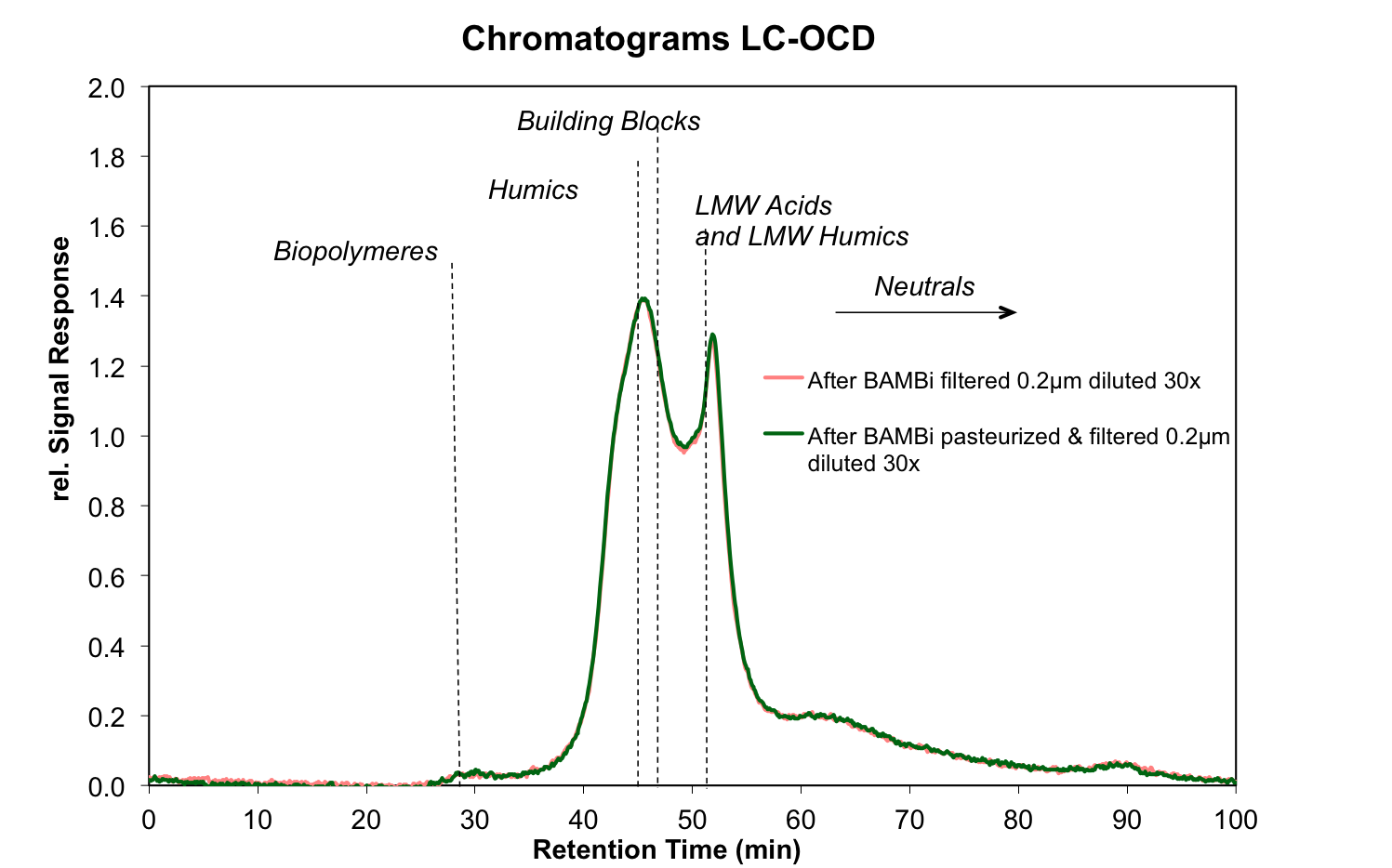


**Figure S1.** Chromatograms from LC-OCD measurements of water after BAMBi before and after pasteurization. Samples were diluted 30 times in order to capture all peaks of the chromatograms.

**

**Figure S2.** Concentration of chlorine produced within 30 min of electrolysis at 0.5 W and 5 W.

**Table S1.** Parameters of the sigmoid function for the S-shaped growth curves of indigenous bacteria, *E. coli,* *P. aeruginosa, Ent. faecalis,* and *S. typhimurium* in water after BAMBi and after BAMBi+GAC.

| **Water** | **Bacteria** | $\boldsymbol{y}_{\boldsymbol{min}}$ | $\boldsymbol{y}_{\boldsymbol{max}}$ | **Net growth (**$\boldsymbol{10}^{\boldsymbol{y}_{\boldsymbol{max}}}\boldsymbol{-}\boldsymbol{10}^{\boldsymbol{y}_{\boldsymbol{min}}}\boldsymbol{)}$ **(CFU/mL)** | $\boldsymbol{logEC}\boldsymbol{50}$ **(h)** | $\boldsymbol{r}_{\boldsymbol{j}}^{\boldsymbol{i}}$ **(h^-1^)** |
| --- | --- | --- | --- | --- | --- | --- |
| After BAMBi | Indigenous | 2.9 | 7.1 | 1.3×10^7^ | 22 | 0.100 |
|  | *E. coli* | 3.8 | 5.6 | 3.9×10^5^ | 14 | 0.100 |
|  | *P. aeruginosa* | 3.5 | 5.9 | 7.9×10^5^ | 12 | 0.130 |
|  | *Ent. faecalis* | 2.9 | 3.8 | 5.5×10^3^ | 12 | 0.010 |
|  | *S. typhimurium* | 3.5 | 4.3 | 1.7×10^4^ | 12 | 0.010 |
| After BAMBi+GAC | Indigenous | 3.3 | 5.6 | 4.0×10^5^ | 16 | 0.080 |
|  | *E. coli* | 3.0 | 3.8 | 5.3×10^3^ | 14 | 0.014 |
|  | *P. aeruginosa* | 3.0 | 4.5 | 3.1×10^4^ | 12 | 0.100 |
|  | *Ent. faecalis* | 2.8 | 3.1 | 6.3×10^2^ | 12 | 0.005 |
|  | *S. typhimurium* | 3.1 | 3.3 | 7.4×10^2^ | 12 | 0.003 |

**Table S2.** Concentration of free and total chlorine in chlorination and electrolysis experiments. Data was shown as mean ± standard error.

| **Experiment** | **Time (min)** | **Free chlorine concentration**  **(mg Cl_2_ L^-1^)** | **Total chlorine concentration**  **(mg Cl_2_ L^-1^)** |
| --- | --- | --- | --- |
| **0.07 mg Cl_2_ L^-1^** | 0 | 0.046 ± 0.003 | 0.088 ± 0.006 |
|  | 5 | 0.021 ± 0.002 | 0.063 ± 0.005 |
|  | 15 | 0.029 ± 0.003 | 0.070 ± 0.008 |
|  | 30 | 0.014 ± 0.003 | 0.056 ± 0.002 |
| **0.14 mg Cl_2_ L^-1^** | 0 | 0.063 ± 0.023 | 0.185 ± 0.059 |
|  | 5 | 0.074 ± 0.017 | 0.162 ± 0.016 |
|  | 15 | 0.056 ± 0.006 | 0.142 ± 0.011 |
|  | 30 | 0.064 ± 0.003 | 0.146 ± 0.005 |
| **0.2 mg Cl_2_ L^-1^** | 0 | 0.100 ± 0.005 | 0.239 ± 0.006 |
|  | 5 | 0.114 ± 0.017 | 0.240 ± 0.009 |
|  | 15 | 0.106 ± 0.003 | 0.220 ± 0.006 |
|  | 30 | 0.076 ± 0.010 | 0.195 ± 0.007 |
| **0.5 mg Cl_2_ L^-1^** | 0 | 0.259 ± 0.011 | 0.464 ± 0.013 |
|  | 5 | 0.201 ± 0.003 | 0.427 ± 0.002 |
|  | 15 | 0.204 ± 0.010 | 0.409 ± 0.014 |
|  | 30 | 0.165 ± 0.009 | 0.392 ± 0.016 |
| **1.7 mg Cl_2_ L^-1^** | 0 | 1.315 ± 0.015 | 1.645 ± 0.055 |
|  | 5 | 0.798 ± 0.008 | 1.285 ± 0.035 |
|  | 15 | 0.503 ± 0.016 | 0.979 ± 0.021 |
|  | 30 | 0.333 ± 0.026 | 0.771 ± 0.017 |
| **3.4 mg Cl_2_ L^-1^** | 0 | 2.985 ± 0.155 | 3.270 ± 0.140 |
|  | 5 | 2.155 ± 0.195 | 2.600 ± 0.180 |
|  | 15 | 1.670 ± 0.230 | 2.105 ± 0.205 |
|  | 30 | 1.330 ± 0.260 | 1.760 ± 0.220 |
| **0.5 W** | 0 | 0.014 | 0.023 |
|  | 5 | 0.281 | 0.451 |
|  | 15 | 0.780 | 0.967 |
|  | 30 | 1.700 | 1.84 |
| **5W** | 0 | 0.015 | 0.021 |
|  | 5 | 0.611 | 0.840 |
|  | 15 | 2.090 | 2.380 |
|  | 30 | 3.400 | 4.100 |

**Figure S3.** Net growth [log_10_(N_t_/N_0_)] of *Ent. faecalis* in the addition of minimal media (Davis), amino acids, and vitamins.
